# Hemorrhagic Renal Cyst, a Case Report

**DOI:** 10.21980/J8C92V

**Published:** 2020-01-15

**Authors:** Mary Rometti, Christopher Bryczkowski, Michael Rohinton Mirza

**Affiliations:** * Rutgers Robert Wood Johnson Medical School, Department of Emergency Medicine, New Brunswick, NJ

## Abstract

**History of present illness:**

A 73-year-old male presented with one day of hematuria associated with urinary frequency and acute on chronic abdominal cramping. On exam, he had diffuse abdominal tenderness, which he stated was normal for him. He was afebrile with no costovertebral angle tenderness or any other pertinent findings on physical exam. The urinalysis showed large red blood cells and small leukocyte esterase and nitrites. Labs were significant for white blood cell count (WBC) 24.6/mm^3^, hemoglobin 11.6 g/dL, blood urea nitrogen (BUN) 56 mg/dL, creatinine 3.8 mg/dL (baseline 2.8 six months ago), glomerular filtration rate (GFR) 16 mL/min. These findings were consistent with acute on chronic kidney injury with concomitant urinary tract infection – specifically concerning for pyelonephritis or an infected renal stone.

**Significant findings:**

Bedside renal ultrasound demonstrated a right renal cyst with echogenic debris consistent with a hemorrhagic cyst (red arrow). In addition, a computed tomography (CT) scan of the abdomen and pelvis revealed a 4mm non-obstructing right renal stone and bilateral renal cysts. The CT also confirmed the ultrasound finding of a right renal cyst with mild perinephric stranding possibly consistent with a hemorrhagic cyst.

**Discussion:**

Simple renal cysts are typically single, unilateral, and usually possess four distinct characteristics: lack internal echoes, have increased posterior acoustic enhancement, have a uniform round/oval shape, and have thin posterior walls/demarcated borders.^1^ If all of these ultrasound features are met, additional imaging does not always have to be obtained.^1^,^2^ Simple renal cysts are usually benign, asymptomatic, and often appear as incidental findings on imaging.^2^,^3^ Generally, the number of renal cysts increase as a person ages.^3^

A renal cyst may be classified as a complex cyst when it fails to be defined as a simple cyst.^1^ Characteristics of complex renal cysts may include septations, calcifications, internal echoes, or other irregularities.^1^ Cysts can also become more complex by hemorrhage or infection, which is usually evident on ultrasound by internal echoes.^1^ Calcifications can also form within the cyst, which can make it challenging to discriminate a simple cyst from cystic renal tumors.^2^ Both malignant and hemorrhagic cysts often have irregular boarders and echogenic material within their walls and within the cyst.^4^ On ultrasound, infected renal cysts are characterized by thickened walls sometimes with debris or gas.^1^,^3^ Calcifications may be present with increased attenuation.^3^ Infected cysts are diagnosed by a combination of imaging findings and clinical characteristics.^3^,^5^ While simple cysts are usually asymptomatic, malignant or more complex cysts are more likely to be symptomatic.^3^

To further distinguish hemorrhagic cysts from malignant tumors, a CT or magnetic resistance imaging (MRI) should be performed.^2^ Computed tomography is more sensitive than ultrasound for identifying a renal mass, but ultrasound is effective for further characterizing a simple cyst from a complex cyst.^3^,^6^ One study reported that CT, MRI, and MRI with diffusion-weighted imaging (DWI) had 100% sensitivity at identifying the presence of possible malignant renal lesions, but CT and MRI had lower specificity (66.9% and 68.8%) than MRI with DWI (93.8%).^7^

Further classifying the type of renal cyst – simple vs complex or hemorrhagic vs infected vs malignant – aids in guiding management. While simple cysts usually do not need additional imaging, complex cysts may need to be further characterized.^2^ If malignancy is unlikely, hemorrhagic cysts are typically followed with serial ultrasounds.^1^ If there is concern for infection, antibiotics should be started.^5^ Further evaluation may include aspiration and drainage.^1^

**Patient Course:**

This patient was started on antibiotics and admitted to the hospital. Urology, nephrology, and infectious disease were consulted. He was continued on antibiotics for 3 weeks due to concern for possible infected renal cyst. The patient was discharged and recommended to follow-up with urology for an outpatient cystoscopy and repeat renal ultrasound in 3 months to evaluate for a possible neoplasm.

**Topics:**

Renal cyst, hemorrhagic cyst, hematuria, bedside ultrasound, POCUS.

[Fig f1-jetem-5-1-v1] [Fig f2-jetem-5-1-v1]

**Figure f1-jetem-5-1-v1:**
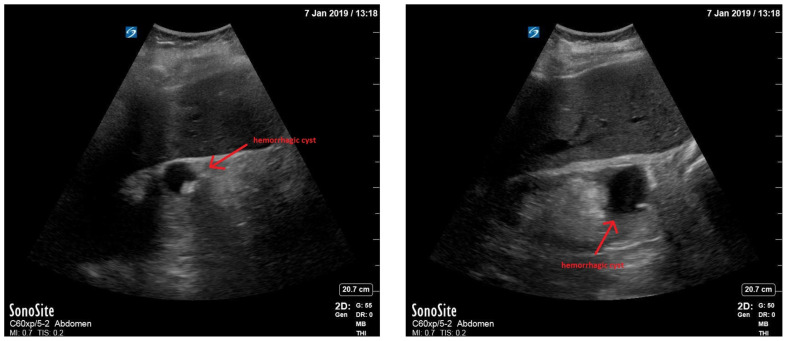


**Figure f2-jetem-5-1-v1:**
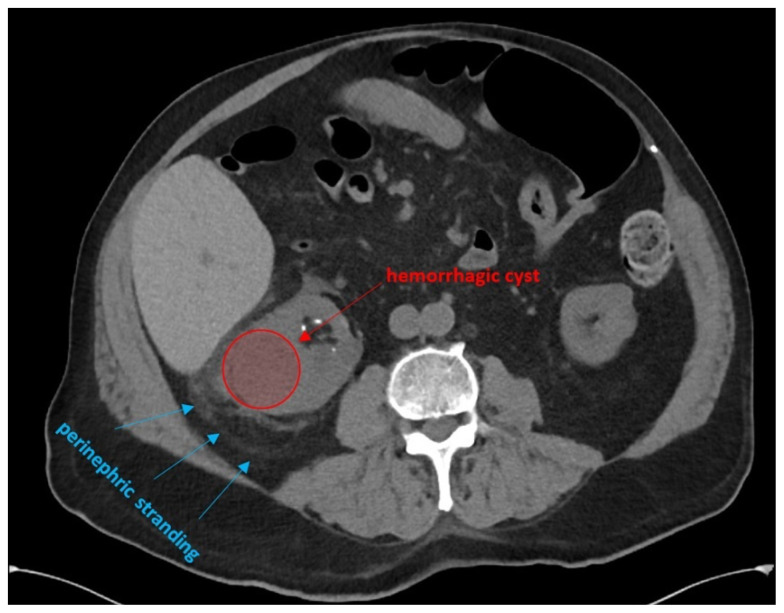


## Supplementary Information














