# Supplementary figures and images for: Hemorrhagic Renal Cyst, a Case Report
Source: J Educ Teach Emerg Med. 2020 Jan 15;5(1):V1–3. doi: 10.21980/J8C92V (PMC10332543; doi:10.21980/J8C92V)

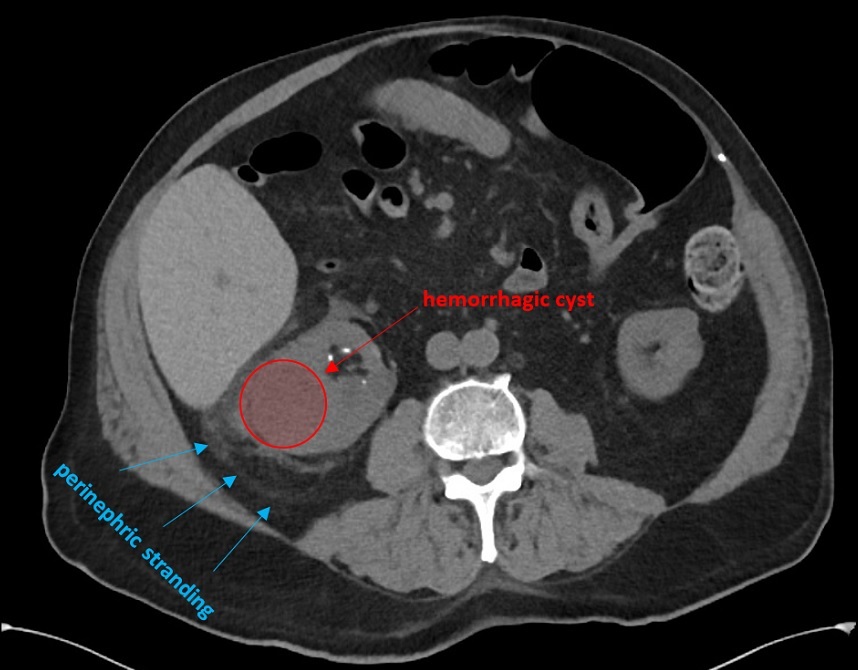

Supplement: Supplementary file 1 [file jetem-5-1-v1-supp1.jpg]

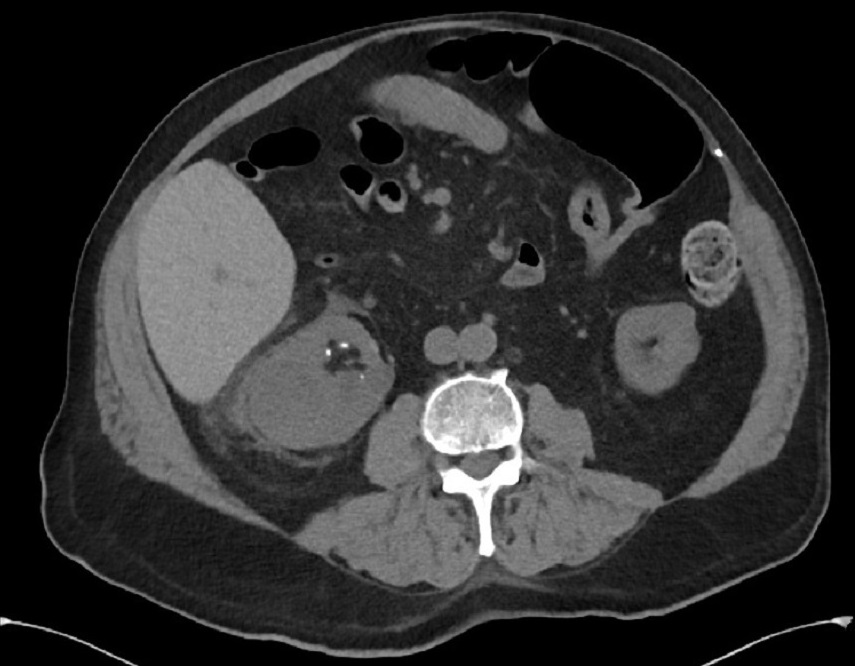

Supplement: Supplementary file 2 [file jetem-5-1-v1-supp2.jpg]

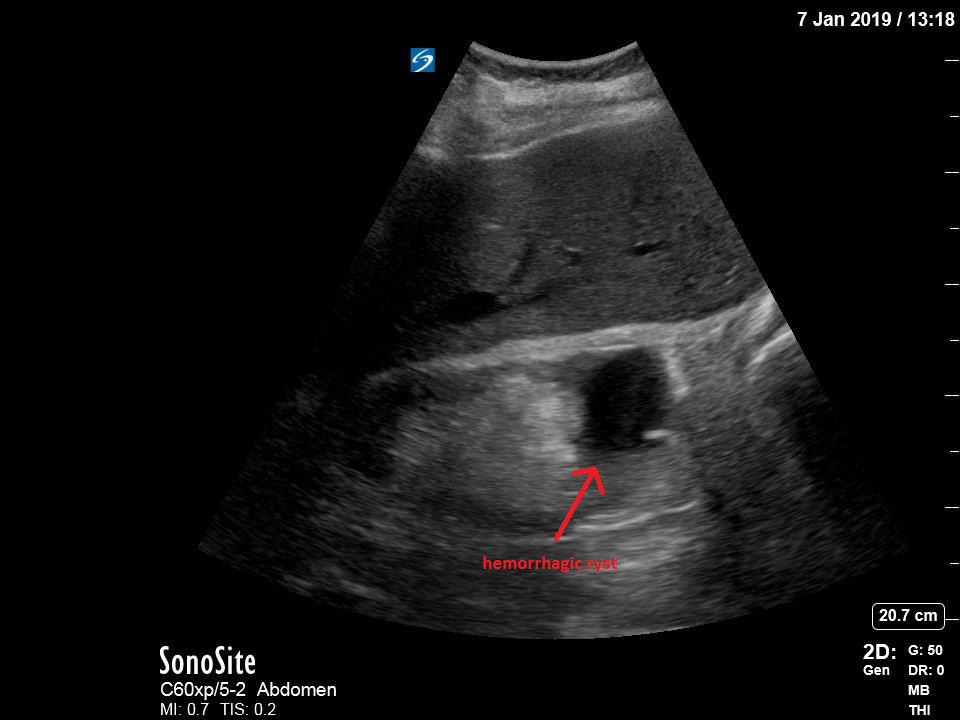

Supplement: Supplementary file 3 [file jetem-5-1-v1-supp3.jpg]

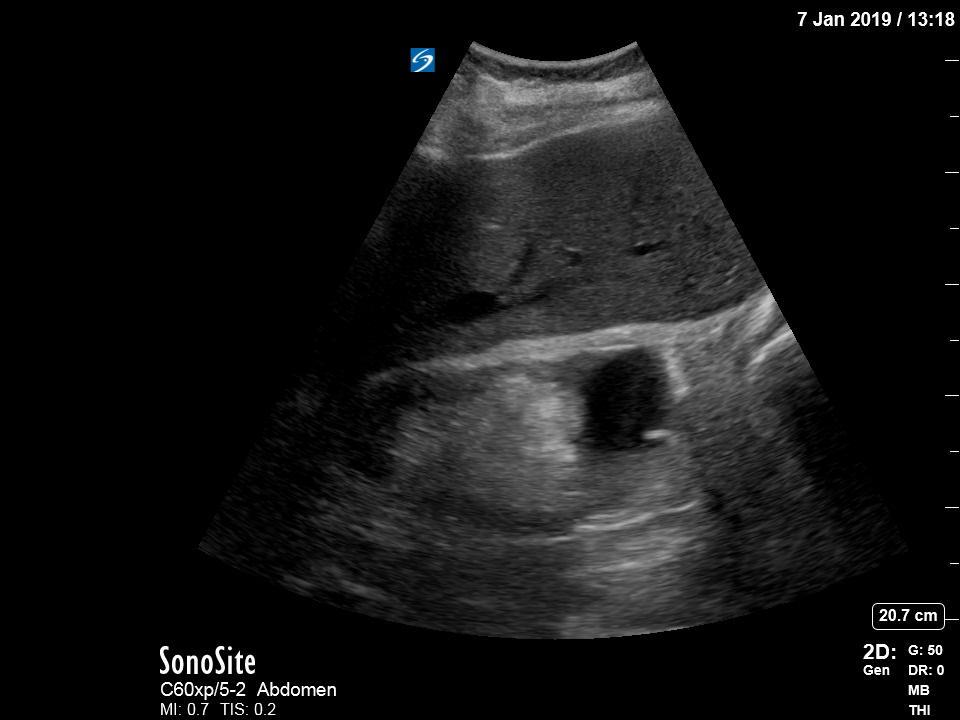

Supplement: Supplementary file 4 [file jetem-5-1-v1-supp4.jpg]

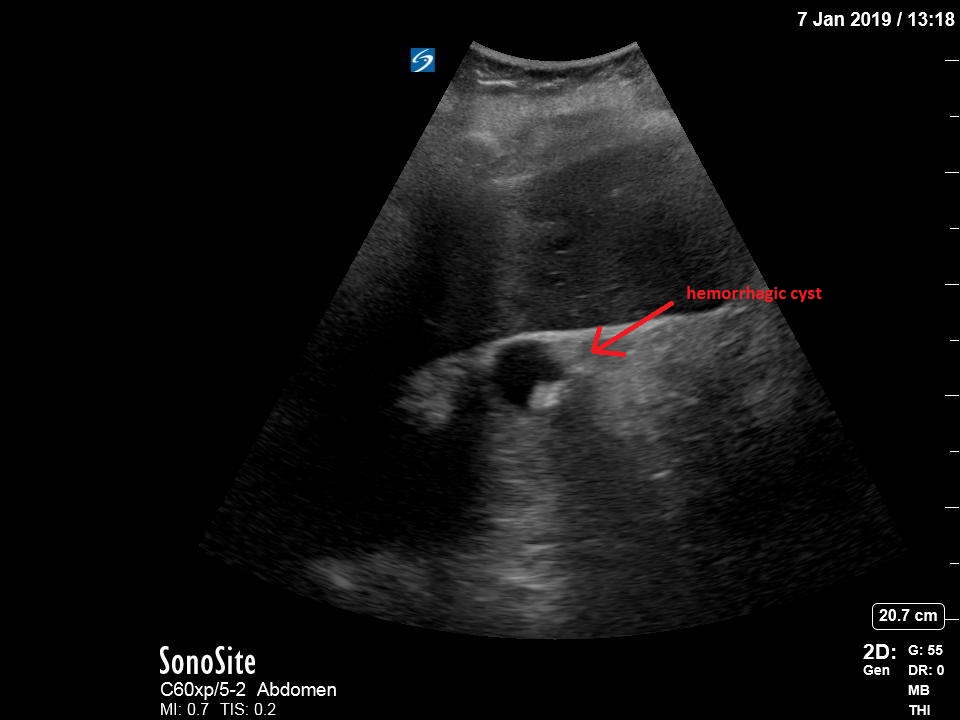

Supplement: Supplementary file 6 [file jetem-5-1-v1-supp6.jpg]

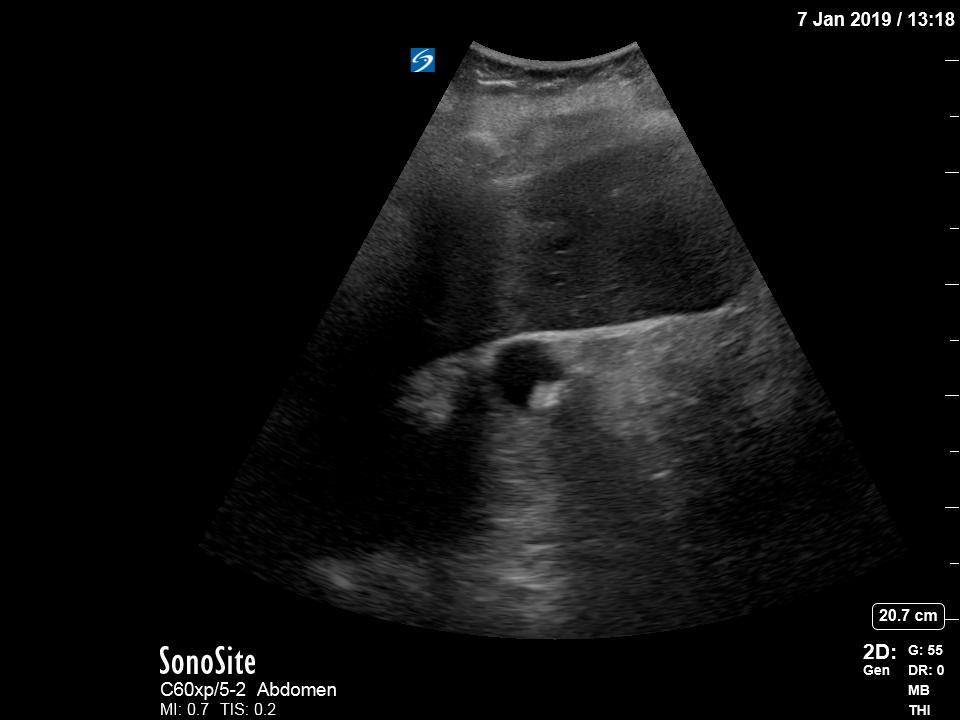

Supplement: Supplementary file 7 [file jetem-5-1-v1-supp7.jpg]
